# Rheology of electromagnetohydrodynamic tangent hyperbolic nanofluid over a stretching riga surface featuring dufour effect and activation energy

**DOI:** 10.1038/s41598-022-18998-9

**Published:** 2022-08-26

**Authors:** Kanayo Kenneth Asogwa, B. Shankar Goud, Nehad Ali Shah, Se-Jin Yook

**Affiliations:** 1 Department of Mathematics, Nigeria Maritime University, Okerenkoko, Delta State Nigeria; 2 Department of Mathematics, JNTUH University College of Engineering Hyderabad, Kukatpally, Hyderabad, Telangana 500085 India; 3 Department of Mechanical Engineering, Sejong University, Seoul, 05006 Republic of Korea; 4 School of Mechanical Engineering, Hanyang University, 222 Wangsimni-ro, Seongdong-gu, Seoul, 04763 Republic of Korea

**Keywords:** Mechanical engineering, Fluid dynamics

## Abstract

The present model deals with the consequence of Dufour, activation energy, and generation of heat on electromagnetohydrodynamic flow of hyperbolic tangent nanofluid via a stretching sheet. This offers a broad significance in several engineering fields. With adequate similarity variables, the regulating governing equations of PDEs are renovated into nonlinear ODEs. The numerical output of the produced ordinary differential equations is conducted with MATLAB bvp4c. The influence of increasing features on temperature, velocity, concentration patterns, drag force coefficient, Sherwood number and Nusselt number is depicted graphically and numerically. Hence, the resultant conclusions are confirmed utilising contrast with earlier output. Interestingly, the activation energy retards the nanofluid's tangential hyperbolic concentration distribution and the rise in temperature of the hyperbolic tangential nanofluid flow is traceable to an increase in the Dufour effect, However, the electromagnetohydrodynamic variable increases the velocity distribution, which influences the Power law index. Conclusively, the rate of heat transfer is inhibited when the thermophoresis parameter, heat source and the Weissenberg number are enhanced.

## Introduction

Heat transmission on non-Newtonian fluid investigations is significant, as the features of a fluid with dispersed nanoparticles cannot be adequately characterised by the Newtonian fluid conception. The study of non-Newtonian materials is relevant to a wide variety of fields. Materials of this kind have found extensive applications in fields as diverse as oil reservoir engineering, biotechnology geophysics, the nuclear and chemical industries, and many more. slurries, Ketchup, aint, paper pulp, polymer solutions, dirt, are just a few examples of non-Newtonian liquids. Considering the size of scientific and industrial progress, researchers are keen to scrutinized the physicochemical approach. The heat transmission flow properties of rheological fluids, in this instance, are critical in food science, fossil fuel extraction, applied physics, medicine, and polymer dissolving sectors. Tangent hyperbolic fluids are non-Newtonian fluids with shear-thinning features. Similarly, a pseudoplastic fluid framework with four characteristics can also describe shear-thinning processes; this type is called hyperbolic tangent fluid. To better understand the behavior of these materials, several models of non-Newtonian liquids have been constructed in the scientific literature. Here's an example: Since its viscosity decreases with increasing shear rate, the tangent hyperbolic liquid may be used as a model to study shear thinning properties. In a porous medium, Reddy et al.^1^ explored the peristaltic transport of a hyperbolic tangent fluid. Hayat et al.^2^ investigated the hydromagnetic flow of a tangential hyperbolic nanofluid formed by an impermeable surface considering Brownian mobility and thermophoresis features. Using the built-in MATLAB bvp4c, Hussain et al.^3^ addressed unsteady MHD flow, including nanoparticles and motile microorganisms, utilising a porous stretchable wedge that has 2nd slip and a Nield threshold. Hayat et al.^4^ addressed hyperbolic tangent fluid flow incorporating Soret-Dufour numbers. Sabu et al.^5^ revealed the significance of nanoparticles’ shape and thermo-hydrodynamic slip constraints on MHD alumina-water nanoliquid flows over a rotating heated disk: the passive control approach. Mahdy and Chamkha^6^ investigated the thermophysical consequences of a time dependent MHD delineation in a permeable medium of tangential hyperbolic nanofluid considering extending wedge using numerical technique. Shafiq et al.^7^ investigated mass and heat transport rates in microorganisms containing hyperbolic tangent nanofluids with MHD and a zero mass flux constraint. Naseer et al.^8^ studied hyperbolic tangent fluid boundary layer in a stretchable longitudinal cylinder. Dawar et al.^9^ studied towards a new MHD non-homogeneous convective nanofluid flow model for simulating a rotating inclined thin layer of sodium alginate-based Iron oxide exposed to incident solar energy. Nadeem et al.^10^ investigated the behaviour of micro hyperbolic tangent liquid in a curved tube.

Generally, boundary layer flows impacted by MHD play a critical role in manufacturing and technical procedures, including the construction of MHD turbines, flow metres, and nuclear reactors. External magnetic fields are widely used to control high conductivity fluid flows, such as semiconductor melting or liquid metals, referred to as conventional MHD flow. This method is ineffective for fluids with low electrical conductivity, such as sea water. A Riga surface generates Lorentz force. Riga refers to a plate surface containing mutually placed magnets and electrodes. This plate is unique because it induces electromagnetic energy sufficient to generate Lorentz forces along the surface, thereby restricting the flow of slightly conducting fluid. The plate was originally constructed from an array of interspaced and obligatory magnets distributed in a spanwise configuration. It can be utilised to prevent boundary layer tearing caused by radiation. In this regard, the Riga plate-induced laminar flow has been examined in physical properties. Gailitis and Lielausis^11^ leveraged the Riga plate for regulating fluid motion. The relevance of chemical changes involving energy activation driving tangent hyperbolic nanofluid Riga wedge flow in the presence of a source of heat was reported by Abdal et al.^12^. They discovered that as the modified Hartmann number escalates from 13.3 to 21.93%, the drag force is significantly increased. Shafiq et al.^13^ scrutinized the heated nanostructures layer by incorporating an electro-magnetic actuator into a Riga surface. Farooq et al.^14^ presented the stagnation point flow through a Riga plate exhibiting chemical interactions. Wakif et al.^15^ addressed the advective EMHD flow behaviour of an electrical current generating fluid across a vertical electromagnetic surface. Hayat et al.^16^ investigated the effect of varied thickness on a stretched electromagnetic plate. Ahmad et al.^17^ investigated the dynamics of convective nanofluid flow over a strongly suctioned Riga surface. Shaw et al.^18^ examined a variable-effects extended Riga surface. Using numerical method, Rafique et al.^19^ examined the stratification flow of micropolar nanofluid across the Riga Plate. Nadeem et al.^20^ studied an exponentially extending Riga plate for the nanofluid domain. Mahdy and Hoshoudy^21^ investigated time-dependent EMHD tangential hyperbolic nanofluids flow across a heated Riga surface with a chemical process. Fatunmbi et al.^22^ investigated the irreversibility of Eyring–Powell non Newtonian nanoliquid flow through a Riga plate. Alotaibi and Rafique^23^ explored the role of microrotation on nanofluid on a Riga surface. Hayat et al.^24^ tackled the rotational flow of nanofluid via a Riga plate. Asogwa et al.^25^ elucidated the importance of ramped energy using Casson fluid over a tilted Riga plate. Ahmad et al.^26^ Executed a numerical analysis of nanofluid flow past a Riga-plate. Recently, Asogwa et al.^27^ dissected the features of alumina and cupric nanoparticles over a rapid Riga surface with thermal dispersion. Other relevant literature of Riga Plate are cited in^28–30^.

The investigation of mass and energy flux occurrences entails the flow being induced by the contrast in densities produced by concentration and temperature variations and substance structure. The Dufour impact is often used to refer to the thermal gradient generated by the solute differential. The Dufour impact governs mixes of hydrocarbons with lesser and intermediate molecular masses. Like petrochemical engineering and seismology research, numerous utilizations are associated with this process. Investigators demonstrated a strong awareness in these two areas, and as a response, they participated in several investigations. For example, Rasool et al.^31^ investigated the role of thermal diffusion and Dufour effect implications on Darcy–Forchheimer circulation of nanoparticles in a stable immiscible state. They demonstrated that the Dufour effect outcome enhances heat transport in the presence of binary reaction. Goud and Reddy^32^ explored the role of thermal diffusion and Dufour number on MHD time dependent flow through a rapidly inclined vertical heated channel heated using Galerkin FEM. They discovered that as the Dufour values rise, friction diminishes. Likewise, by employing the Galerkin finite element method. Kumar et al.^33^ explored unsteady MHD free convection combining thermal diffusion and Dufour impact phenomena over a vertically fixed surface. Abdelraheem and El-Sapa^34^ addressed MHD nanofluid convection through a squared cavity. They incorporated dual rotation between an external rotating disc and an inside squared form with thermal diffusion and Dufour phenomena. Asogwa et al.^35^ explored thermal distribution and Duffour's effects on non-Newtonian Casson fluid in a permeable medium with heat absorption. Using the perturbation approach, Uwanta et al.^36^ investigated Magnetohydrodynamic impact across a flat channel incorporating Dufour and Soret effects. Some interested results are presented in^37–39^.

Stimulated by the aforementioned literature, the existing research examines patterns of hyperbolic tangent nanofluid across a radiative Riga stretching surface with Dufour effect, heat generation, and activation energy. Here, extensive mathematical transformation is done, followed by computations using the MATLAB bvp4c procedure. The significance of developed variables in the velocity, heat, and concentration domains is illustrated and discussed graphically. The findings may find application in low-density heat exchangers and temperature transmission devices.

## Formulation of the problem

**Figure 1:**
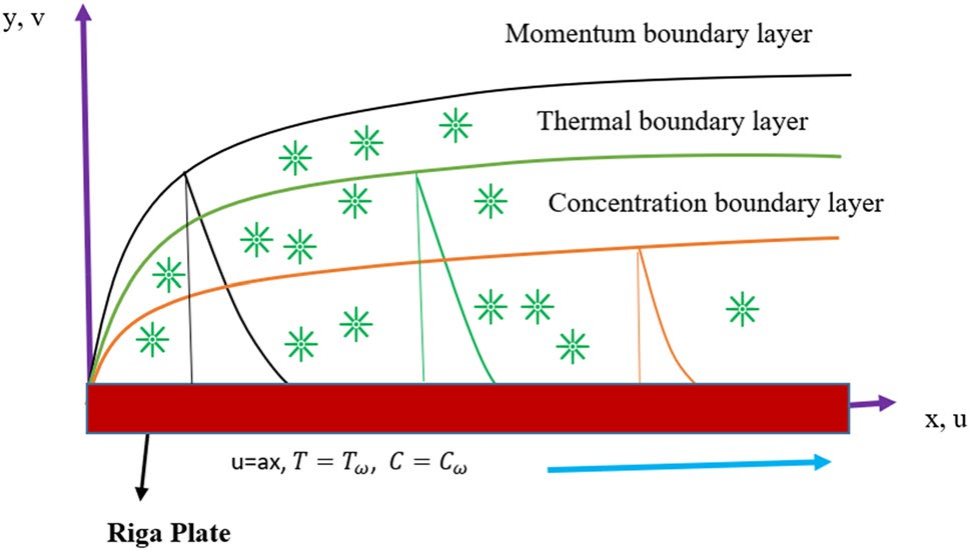
Plate model.

Considering the constant wall thermal performance and concentration with a velocity $u = ax$ along the boundary layer area due to an electrically charged tangential hyperbolic nanofluid flow across a stretched Riga wall, changing thickness and momentum are formulated. Furthermore, the feature of activation energy and heat generation is utilised. Thermophoresis and Brownian motion are used to demonstrate the behaviour of nanofluids. The configuration flow over a Riga tray model is seen in Fig. 1.

A Riga surface denotes magnets and electrodes arranged interdependently along the x-axis and perpendicular to the y-axis. This electromagnetic field can be characterized by the Grinberg concept as $F = \frac{{\pi J_{0} m_{0} e^{{ - \frac{\pi }{l}y}} }}{8}$. In addition, the flow of 2-dimensional tangent hyperbolic nanofluid EMHD across a stretchable Riga wall experienced diffusion-thermo, nonlinear thermal radiation, heat generation, and activation energy in this research analysis.

The governing equations are modeled as follows: Hayet et al.^2^, Waqas et al.^5^, Rasool et al.^31^

1 $$\frac{\partial u}{{\partial x}} + \frac{\partial u}{{\partial y}} = 0,$$

2 $$u\frac{\partial u}{{\partial x}} + v\frac{\partial u}{{\partial y}} = \nu \left( {1 - n} \right)\frac{{\partial^{2} u}}{{\partial y^{2} }} + \sqrt 2 \nu n\Gamma \left( {\frac{\partial u}{{\partial y}}} \right)\frac{{\partial^{2} u}}{{\partial y^{2} }} + \frac{{\pi J_{0} m_{0} }}{{8\rho_{f} }}\exp \left( { - \frac{\pi }{l}} \right)y,$$

3 $$u\frac{\partial T}{{\partial x}} + v\frac{\partial T}{{\partial y}} = \alpha_{f} \frac{{\partial^{2} T}}{{\partial y^{2} }} + \psi \left[ {D_{B} \frac{\partial C}{{\partial y}}\frac{\partial T}{{\partial y}} + \frac{{D_{T} }}{{T_{\infty } }}\left( {\frac{\partial T}{{\partial y}}} \right)^{2} } \right] - \frac{1}{{\left( {\rho C_{p} } \right)_{f} }}\frac{{\partial q_{r} }}{\partial y} + \frac{{Q_{1} }}{{\left( {\rho C_{p} } \right)_{f} }}\left( {T - T_{\infty } } \right) + \frac{{D_{m} K_{T} }}{{C_{s} C_{p} }}\frac{{\partial^{2} C}}{{\partial y^{2} }},$$

4 $$u\frac{\partial C}{{\partial x}} + v\frac{\partial C}{{\partial y}} = D_{B} \frac{{\partial^{2} C}}{{\partial y^{2} }} + \frac{{D_{T} }}{{T_{\infty } }}\frac{{\partial^{2} T}}{{\partial y^{2} }} - K\left( {\frac{T}{{T_{\infty } }}} \right)^{m} (C - C_{\infty } )\exp \left( {\frac{{ - E_{1} }}{KT}} \right).$$

The corresponding associated conditions are as follows:

5 $$\left. {\begin{array}{*{20}l} {u = 0,\quad v = 0,} \hfill & {T = T_{\infty } ,} \hfill & {\quad C = C_{\infty } ,} \hfill & {\quad \forall } \hfill & {y \ge 0,} \hfill \\ {u = ax,} \hfill & {T = T_{\omega } ,} \hfill & {\quad C = C_{\omega } ,} \hfill & {\quad as} \hfill & {y = 0,} \hfill \\ {u \to U_{\infty } ,} \hfill & {T \to T_{\infty } ,} \hfill & {\quad C \to C_{\infty } ,} \hfill & {\quad as} \hfill & {y \to \infty .} \hfill \\ \end{array} } \right\}.$$

The Rosseland approximation is incorporated as

6 $$q_{r} = - \frac{{4\sigma^{*} }}{{3k^{*} }}\frac{{\partial T^{4} }}{\partial y}.$$

Suppose the temperature variations are relatively minimal such that $T^{4}$ could be broadened in a Taylor expansion about $T_{\infty }$ and the elevated terms are omitted, the result is

7 $$T^{4} = 4TT_{\infty }^{3} - 3T_{\infty }^{4} .$$

Equations (6 and 7) develop into

8 $$\frac{{\partial q_{r} }}{\partial y} = - \frac{{16\sigma^{*} T_{\infty }^{3} }}{{3k^{*} }}\frac{{\partial^{2} T}}{{\partial y^{2} }}.$$

The non-dimensional quantities are implemented:

9 $$\left. {\begin{array}{*{20}l} {u = ax\frac{\partial f}{{\partial \zeta }},\;\;v = - \left( {av_{f} } \right)^{\frac{1}{2}} f(\zeta ),\;\;\zeta = \sqrt {\frac{a}{{v_{f} }}} y,\;\;M = \frac{{\pi J_{0} m_{0} v_{f} }}{{8\rho u_{\infty }^{2} }},\;\;S = \sqrt {\frac{{\pi^{2} v_{f} }}{{l^{2} a}}} ,} \hfill \\ {\Theta (\zeta ) = \frac{{T - T_{\infty } }}{{T_{\omega } - T_{\infty } }},\;\;C(\zeta ) = \,\frac{{C - C_{\infty } }}{{C_{\omega } - C_{\infty } }},\;\;Sc = \frac{{v_{f} }}{D},\;\;\Pr = \frac{{v_{f} \rho C_{p} }}{k},\;\;R = \frac{{4\sigma^{*} T_{\infty }^{3} }}{{kk^{*} }},\;\;\psi = \frac{{(\rho C_{p} )_{p} }}{{(\rho C_{p} )_{f} }}.} \hfill \\ \end{array} } \right\}.$$

The non-dimensional components of Eq. (9) are swapped into Eqs. (2), (3), (4) and (5) taking into consideration Eq. (8) producing:

10 $$\left( {\left( {1 - n} \right) + nWef^{\prime\prime}(\zeta )} \right)f^{\prime\prime\prime}(\zeta ) + f(\zeta )f^{\prime\prime}(\zeta ) - \left( {f^{\prime}(\zeta )} \right)^{2} + M\exp ( - S\zeta ) = 0,$$

11 $$\left( {1 + \frac{4}{3}R} \right)\Theta^{\prime\prime}(\zeta ) + \Pr [Nb\Theta^{\prime}(\zeta )C^{\prime}(\zeta ) + Nt\left( {\Theta^{\prime}(\zeta )} \right)^{2} + f(\zeta )\Theta^{\prime}(\zeta ) + Q\Theta (\zeta ) + DuC^{\prime\prime}(\zeta )] = 0,$$

12 $$C^{\prime\prime}(\zeta ) + \frac{{N_{t} }}{{N_{b} }}\theta^{\prime\prime}(\zeta ) + Scf(\zeta )C^{\prime}(\zeta ) - ScK_{1} \left( {1 + \delta \Theta } \right)^{m} \exp \left( {\frac{{ - E_{a} }}{1 + \delta \Theta }} \right)C(\zeta ) = 0.$$

The resulting conditions are as follows:

13 $$\left. {\begin{array}{*{20}l} {f^{\prime}(\zeta ) = 1,\;f(\zeta ) = 0,} \hfill & {\Theta (\zeta ) = 1,} \hfill & {\quad C(\zeta ) = 1,} \hfill & {\quad for} \hfill & {\zeta = 0,} \hfill \\ {f^{\prime}(\zeta ) \to 0,\,} \hfill & {\Theta (\zeta ) \to 0,} \hfill & {\quad C(\zeta ) \to 0,} \hfill & {\quad as} \hfill & {\zeta \to \infty .} \hfill \\ \end{array} } \right\}.$$

where $$Nt = \,\frac{{\psi D_{T} \left( {T_{\omega } - T_{\infty } } \right)}}{{T_{\infty } v_{f} }}$$, $$Nb = \,\frac{{\psi D_{B} \left( {C_{\omega } - C_{\infty } } \right)}}{{v_{f} }}$$, $$E_{a} = \frac{{E_{1`} }}{{KT_{\infty } }}$$, $$Du = \frac{{D_{m} k_{T} (C_{\omega }^{{}} - C_{\infty }^{{}} )}}{{\nu_{f} C_{s} C_{p} (T_{\omega }^{{}} - T_{\infty }^{{}} )}}\,$$, $$\,\,\,Q = \frac{{Q_{1} }}{{a\left( {\rho C_{p} } \right)_{f} }}\,$$, $$We = \frac{{2^{{\tfrac{1}{2}}} a^{{\tfrac{3}{2}}} x}}{{\sqrt {v_{f} } }}\Gamma$$, $$K_{1} = \frac{K}{a}\,$$, $$\delta = \frac{{T_{\omega }^{{}} - T_{\infty }^{{}} }}{{T_{\infty }^{{}} }}\,.$$

The skin friction coefficient, which is an essential boundary layer property, is given by

${C}_{f}=\frac{{\tau }_{w}}{\rho {u}_{w}^{2}}$, ${\tau }_{w}={\left[\left(1-n\right)\frac{\partial u}{\partial y}+\frac{n\Gamma }{\sqrt{2}} {\left(\frac{\partial u}{\partial y}\right)}^{2}\right]}_{\zeta =0}$ and the dimensionless form is expressed as

$$C_{f} \sqrt {{\text{Re}}_{x} } = \frac{n}{2}We(f^{\prime\prime}(0))^{2} + (1 - n)f^{\prime\prime}(0).$$

Nusselt's number is denoted by $N{u}_{x}=\frac{x{q}_{w}}{k({T}_{w}-{T}_{\infty })} f$ or the present study, the local heat flux ${q}_{w}$ at the wall is defined as ${q}_{w}=-{\left[k\left(1+\frac{16{\sigma }^{*}{T}_{\infty }}{3k{k}^{*}}\right)\frac{\partial T}{\partial y}\right]}_{\zeta =0}$.

The local Nusselt number in dimensionless form is given by

$$N{u}_{x}{/\sqrt{Re}}_{x}=-\left(1+\frac{4}{3}Rd\right){\Theta }{^{\prime}}\left(0\right).$$

The Sherwood number is defined as $Sh=\frac{x{j}_{w}}{{D}_{B}}.$ For this study, the local mass flux ${j}_{w}$ is given by ${j}_{w}=-{D}_{w}{\left(\frac{\partial C}{\partial y}\right)}_{\zeta =0}$ also, dimensionless form is given by $\frac{Sh}{{\sqrt {{\text{Re}}_{x} } }} = - C^{\prime}(0)$.

Where the local Reynolds number is ${\text{Re}}_{x} = \frac{{ax^{2} }}{{v_{f} }}.$

## Method of solution

The reduced differential Eqs. (10)–(13) are numerically solved together with Neumann boundary conditions using the bvp4c approach for various parameter values.

**Figure 2:**
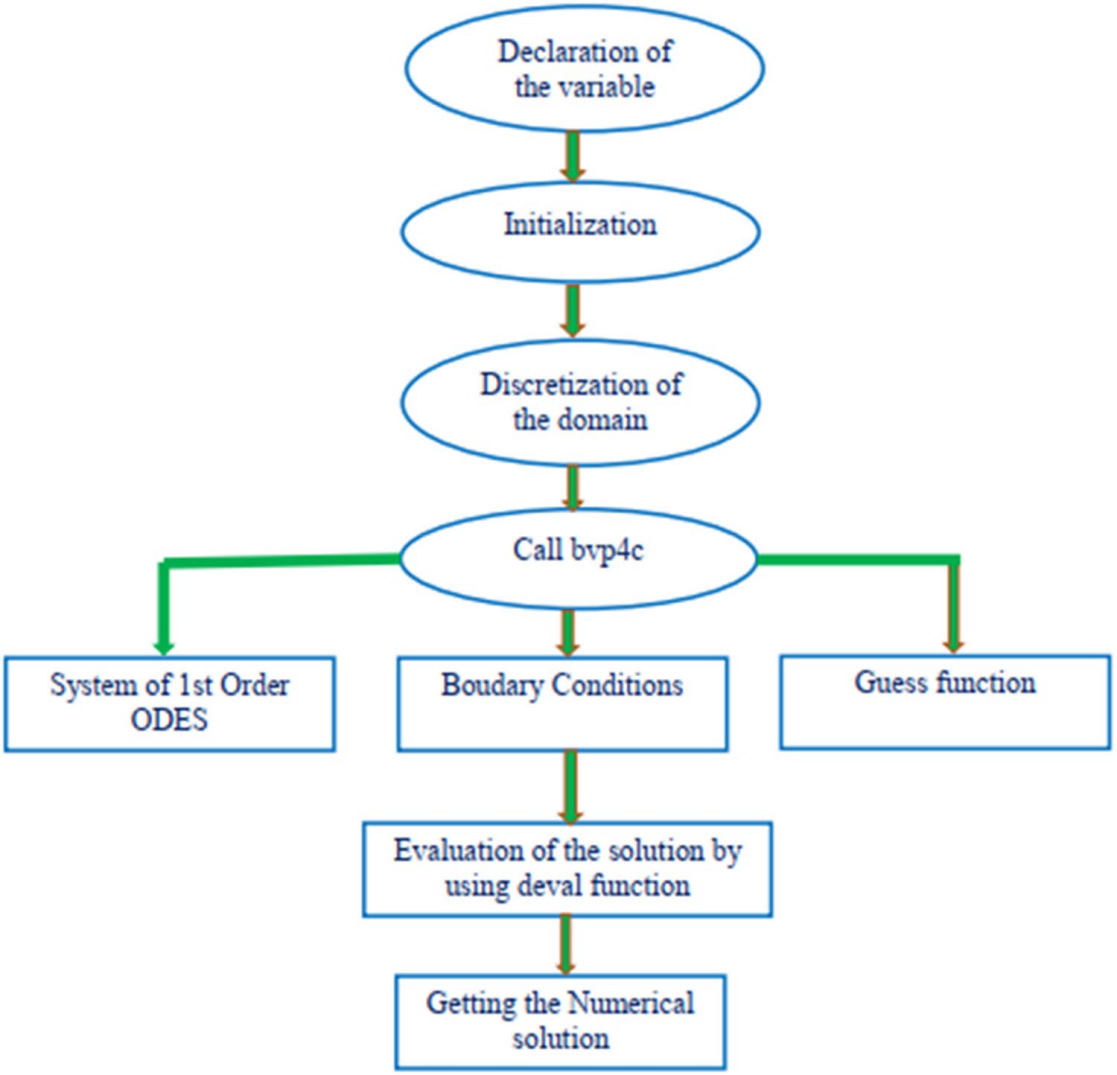
Flow chart solution.

Using Matlab's bvp4c solver, which adopts a finite difference strategy. Before MATLAB bvp4c can be used, the Eqs. (10)–(13) must be transformed into a system of first-order equations. The systematic way for the solution follows according to Fig. 2.

Just let $\xi ={\left[f \; {f}{^{\prime}} \; {f}{^{\prime\prime} } \; \Theta \; \Theta ^{\prime } \; C \; C{^{\prime}} \right]}^{T}.$ which gives

*Step 1* We now have a system of equations of the first order.:

$$\frac{d}{d\zeta }\left(\begin{array}{c}\xi \left(1\right)\\ \xi \left(2\right)\\ \xi \left(3\right)\\ \xi \left(4\right)\\ \xi \left(5\right)\\ \xi \left(6\right)\\ \xi \left(7\right)\end{array}\right)=\left(\begin{array}{c}\xi \left(2\right)\\ \xi \left(3\right)\\ -\left(f{f}^{^{\prime\prime} }-{\left({f}^{^{\prime}}\right)}^{2}+M{e}^{-S\zeta }\right)/(\left(1-n\right)+nWef\left(3\right))\\ \xi \left(5\right)\\ -\left({N}_{b}\Theta ^{\prime } {C}^{{\prime}}+{N}_{t}{\left(\Theta ^{\prime } \right)}^{2}+f\Theta ^{\prime } +Q\Theta ^{\prime } +\mathrm{Du}f{^{\prime}}\left(7\right)\right)/\left(1+\frac{4R}{3}\right)\\ \xi \left(7\right)\\ -\left(\frac{{N}_{t}}{{N}_{b}}{} \Theta ^{\prime \prime } +Scf{C}^{^{\prime}}+Sc{K}_{1}{\left(1+delta \; \Theta ^{\prime } \right)}^{m}{e}^{\frac{-{E}_{a}}{1+\delta }}C\right)\end{array}\right)$$

*Step 2* The numerical solution is performed using the in-built bvp4c MATLAB solver, boundary conditions, and an appropriate finite value for the far-range boundary condition. The significance of the boundary values as $\eta \to \infty$ say $\eta \to 10$.

*Step 3* Initial criteria that apply are as follows:

$$\left(\begin{array}{c}\xi a\left(1\right)\\ \xi a\left(2\right)\\ \xi a\left(4\right)\\ \xi a\left(6\right)\\ \xi b\left(2\right)\\ \xi b\left(4\right)\\ \xi b\left(6\right)\end{array}\right)=\left(\begin{array}{c}0\\ 1\\ 1\\ 1\\ 0\\ 0\\ 0\end{array}\right)$$

The scaling factor is marked by = 0.01, and the convergence requirements are specified to the fifth decimal place.

When Matlab bvp4c is used, just three items are required to solve the bvp.

- A function ODEs for evaluating ordinary differential equations.
- A function called BCs (Boundary conditions) calculates the boundary condition's residual.
- A solit structure that contains both a mesh estimation and a mesh solution. In Matlab, ODEs are treated in a manner similar to IVP solvers.

## Results and discussion

The numerical solution of the set of ODEs generated from the momentum, energy, and concentration Eqs. (10)–(13) and subjected to the boundary conditions was accomplished with the help of the bvp4c function from a MATLAB Software. The beauty of MATLAB bvp4c is that it is numerically more stable and converges more quickly. We got velocity, concentration, and temperature graphs for several values of the controlling parameters. The findings are shown graphically.

**Figure 3:**
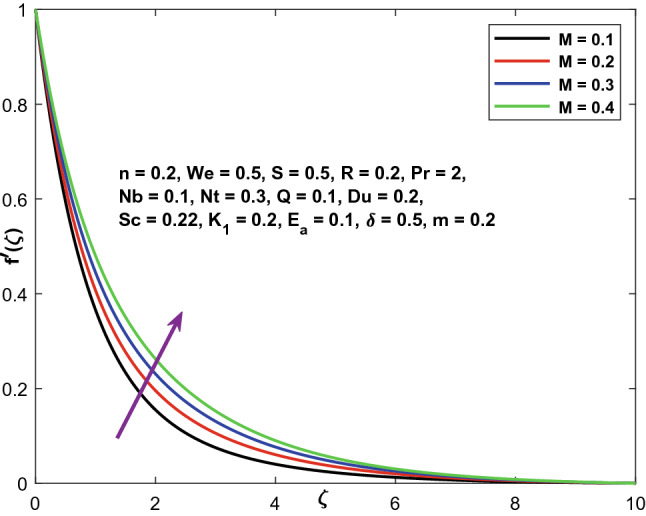
Character of $M$ versus ${f}^{^{\prime}}\left(\zeta \right)$.

**Figure 4:**
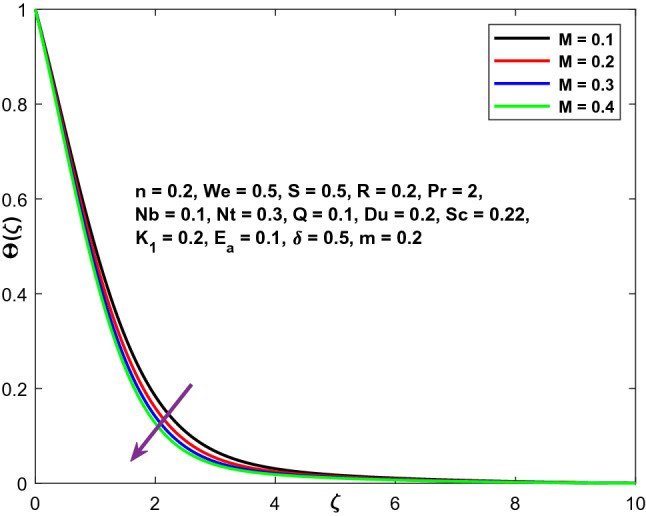
Character of $M$ versus $\Theta \left(\zeta \right)$.

**Figure 5:**
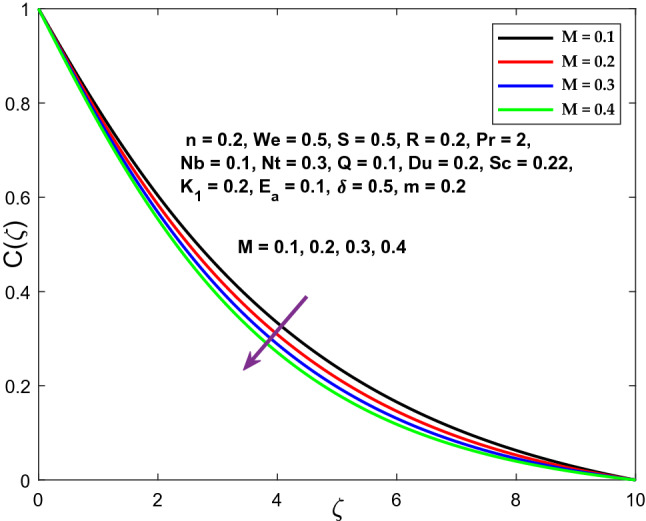
Character of $M$ versus $C\left(\zeta \right)$.

The velocity, temperature, and concentration profiles are displayed in Figs. 3, 4 and 5 to demonstrate the controllable effect of modified Hartmann number (M). The modified Hartmann number $\left(M\right)$ increases the velocity distribution and reduces the temperature, concentration distribution in the data shown in Fig. 3, 4 and 5. Increased M estimates increase the magnitude of the external electric field that extends beyond the usual dimension, resulting in the formation of wall parallel Lorentz force. The velocity distribution advances in a linear fashion.

**Figure 6:**
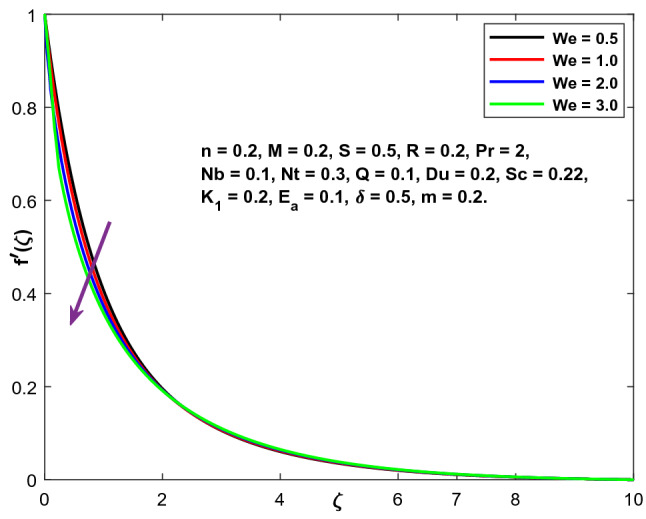
Character of $We$ versus $f{^{\prime}}\left(\zeta \right)$.

**Figure 7:**
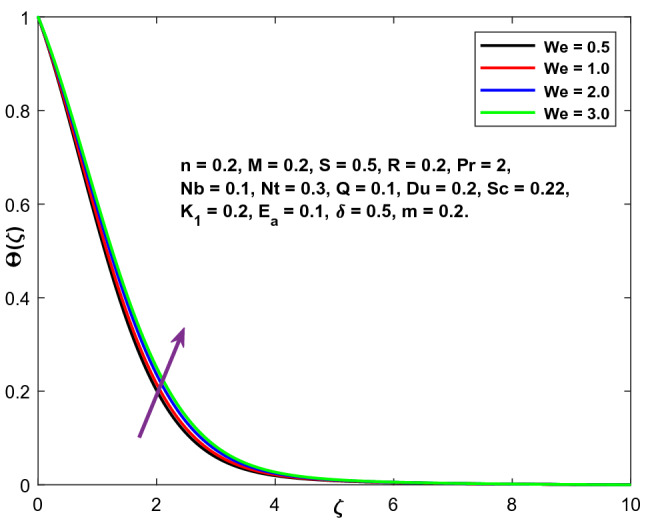
Character of $We$ versus $\Theta \left(\zeta \right)$.

**Figure 8:**
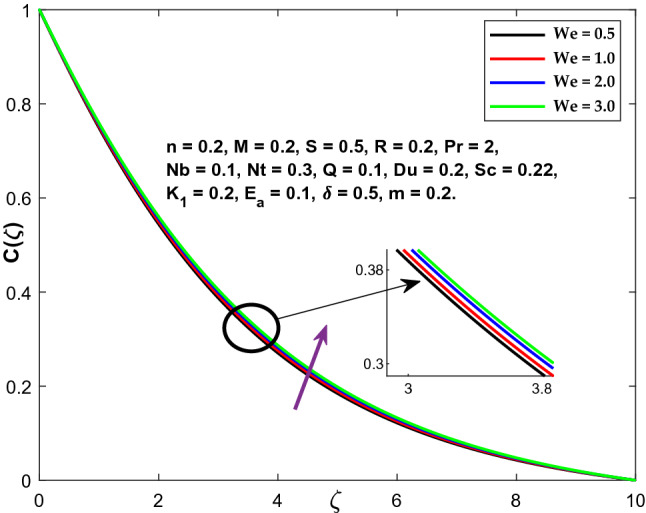
Character of $We$ versus $C\left(\zeta \right)$.

The impacts of the emerging physical factor, i.e., The Weissenberg number's consequences on the fluid velocity, temperature, and concentration areas, are shown in Figs. 6, 7 and 8. Figure illustrates the relationship between the fluid velocity, the fluid temperature, and the concentration. The velocity profiles are seen to be diminishing functions of (We). The Weissenberg value expresses relaxation time's proportion (ratio) to the duration required for a certain procedure. Increasing (We) reduces the particular process time, which results in a reduction in both the velocity component and thickness of the boundary layer. By increasing the value of (We), the fluid concentration and temperature profiles are enhanced.

**Figure 9:**
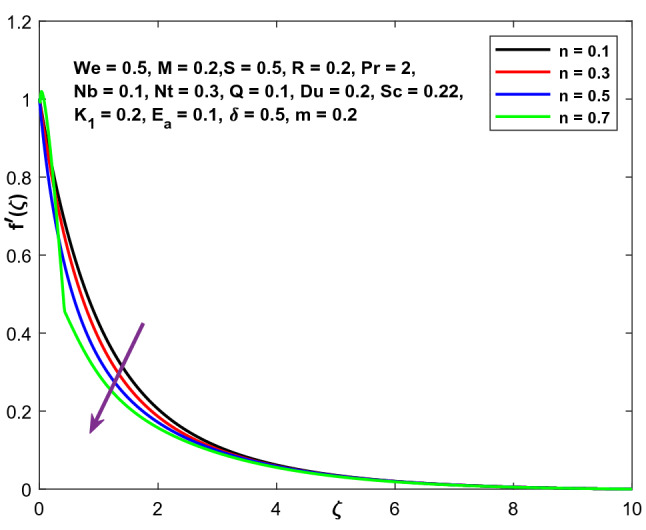
Character of $n$ versus $f{^{\prime}}\left(\zeta \right)$.

**Figure 10:**
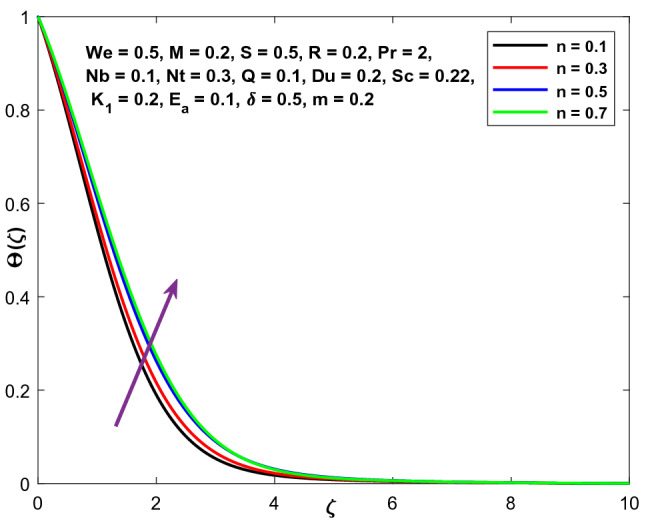
Character of $n$ versus $\Theta \left(\zeta \right)$.

**Figure 11:**
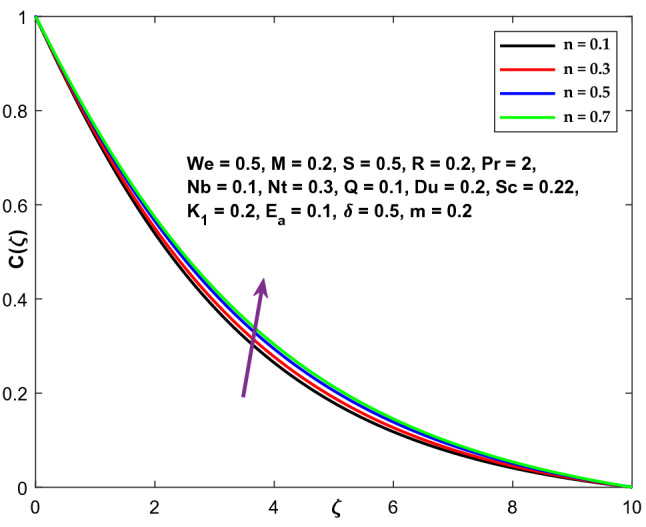
Character of $n$ versus $\mathrm{C}\left(\zeta \right)$.

Figures 9, 10 and 11 illustrate the variations in the velocity, temperature, and concentration domains generated by the power-law index n. The impact of the power-law index n on the velocity distribution is seen in Fig. 9. The dimensionless velocity declines as the power-law index n increases. The temperature and concentration fields are shown in Figs. 10 and 11 as they vary as a function of n. A spike in the power-law coefficient (n) leads to a rise in the fluid's viscosity. The velocity of the fluid reduces as a consequence, while the temperature and concentration fields improve.

**Figure 12:**
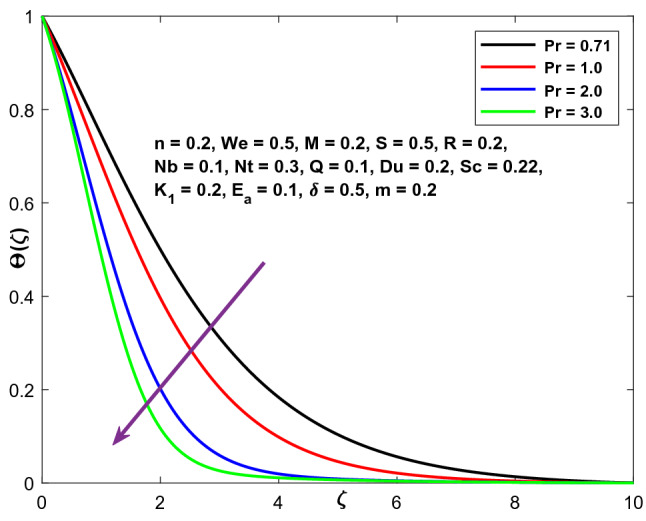
Character of $Pr$ versus $\Theta \left(\zeta \right)$.

Figure 12 depicts the function of Pr on temperature. Prandtl number (Pr) controls the thermal pattern in the figure. The curves in this figure illustrate that a rise in Pr translates into a drop in the energy profile. This is because heat conductivity diminishes as Pr increases. Physically, a high Pr value indicates a poor thermal conductivity, which diminishes conduction and consequently the thermal boundary layer, resulting in a fall in fluid temperature.

**Figure 13:**
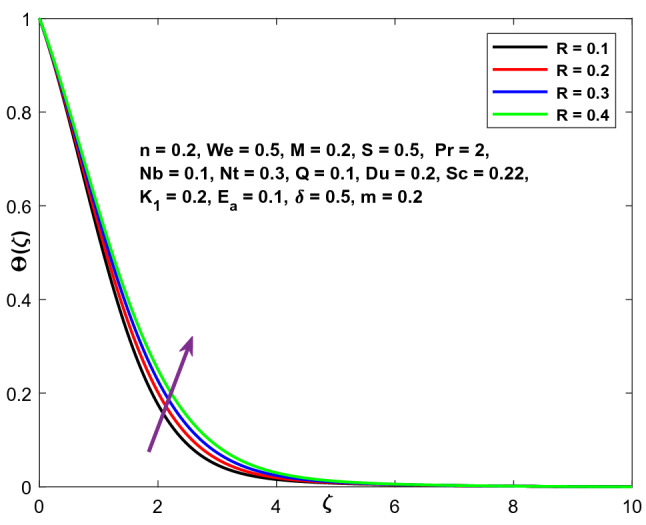
Character of $R$ versus $\Theta \left(\zeta \right)$.

Figure 13 exhibits the function of the radiation parameter (R) on the temperature field. It is noticed that when R grows, the temperature distribution improves dramatically, as an upsurge in the radiation parameter transmits additional heat to the fluid, resulting in an increase in the temperature and structural thickness of the boundary layer.

**Figure 14:**
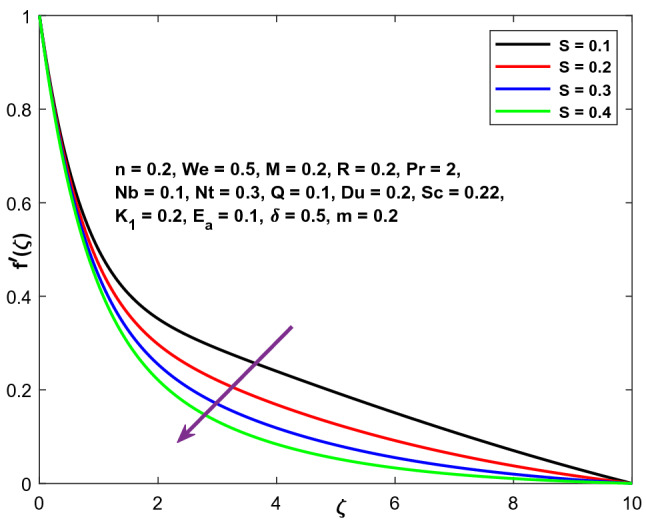
Character of $S$ versus $f{^{\prime}}\left(\zeta \right)$.

**Figure 15:**
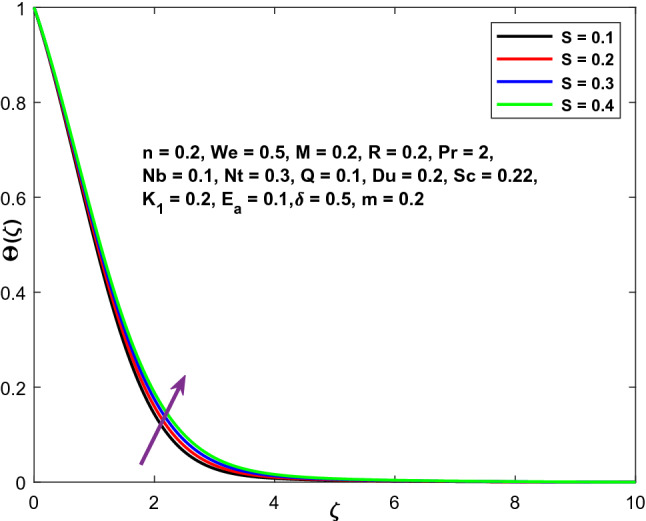
Character of $S$ versus $\Theta \left(\zeta \right)$.

**Figure 16:**
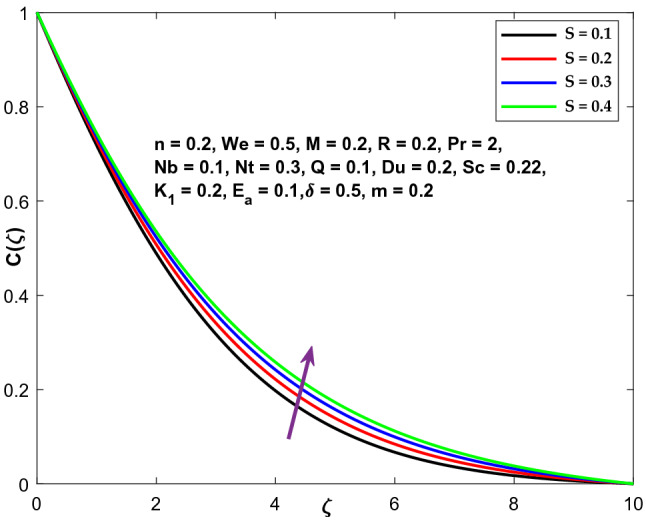
Character of $S$ versus $\mathrm{C}\left(\zeta \right)$.

The impact of decreasing parameter S is seen in Figs. 14, 15 and 16, whereas the thermal and concentration curves exhibit the opposite effect. Internal forces inside the thick wall rise as Nb increases, resulting in a decrease in the momentum boundary layer and flow velocity. The stretching velocity decreases as the wall thickness factor increases. Due to the fact that this is primarily concerned with the asymptotic behaviour of the velocity distribution, increasing the wall thickness factor raises the liquid velocity monotonically.

**Figure 17:**
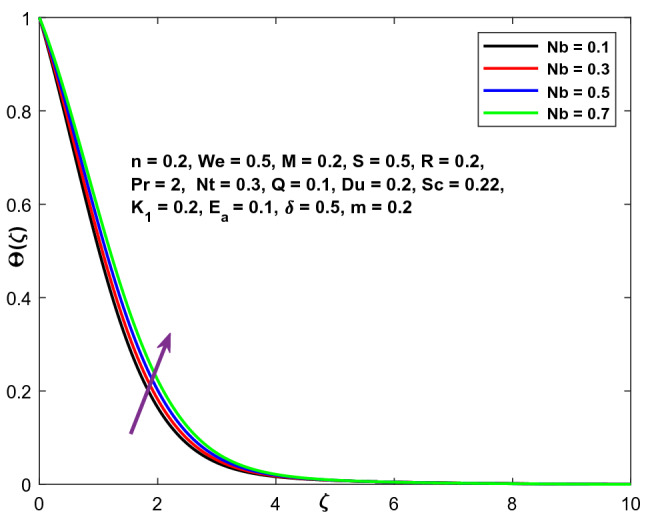
Character of $Nb$ versus $\Theta \left(\zeta \right)$.

**Figure 18:**
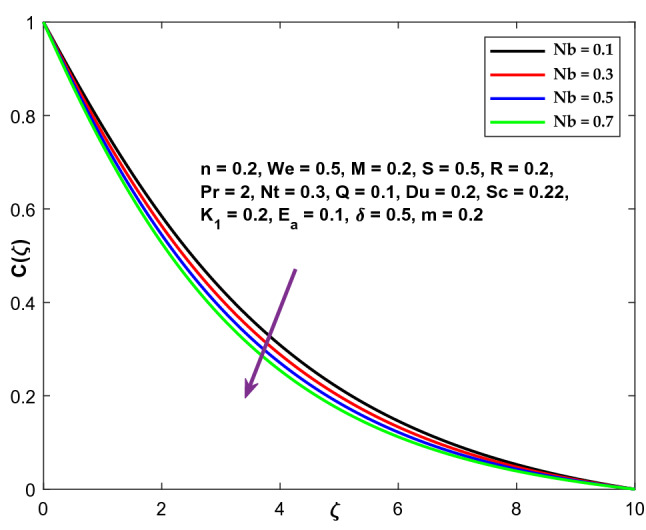
Character of $Nb$ versus $\mathrm{C}\left(\zeta \right)$.

Figure 17 highlights the function of Brownian motion coefficient Nb on the temperature variation. The higher temperature distribution is obtained when the Brownian motion coefficient is enhanced. Consequently, the thickness of the thermal boundary layer grows. As the Brownian motion parameter improves, the random motion of the fluid particles increases, resulting in increasing heat output. As a result, temperature distribution improves. The concentration profile exhibits the inverse phenomena in Fig. 18.

**Figure 19:**
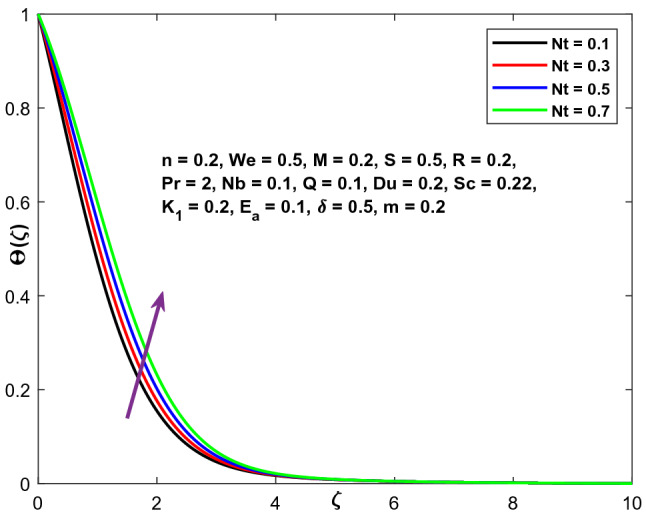
Character of $Nt$ versus $\Theta \left(\zeta \right)$.

Figure 19 illustrates the impact of the thermophoresis parameter Nt on the temperature gradient. For larger values of Nt, both temperature and thermal boundary layer width exhibit dominant behaviour. The strategy of Thermophoresis is a technique by which particles heated are drawn from a hot surface toward a cooler location. As a result, the temperature of the fluid improves.

**Figure 20:**
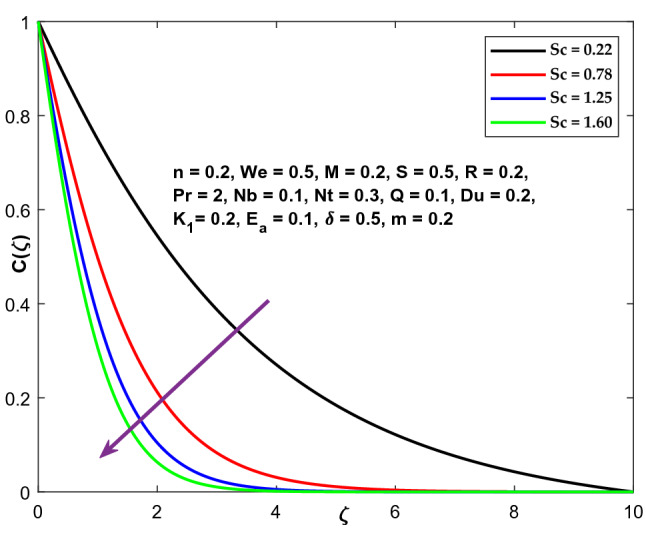
Character of $Sc$ versus $\mathrm{C}\left(\zeta \right)$.

Figure 20 exhibits the Schmidt number's (Sc) trend on concentration curvatures. It examines the relative efficacy of momentum and mass transmission through diffusion within the hydrodynamic (velocity) and chemical (species) boundary surfaces. Increased Schmidt coefficient reduces the fluid's mass diffusivity, associated with decreased concentration profiles.

**Figure 21:**
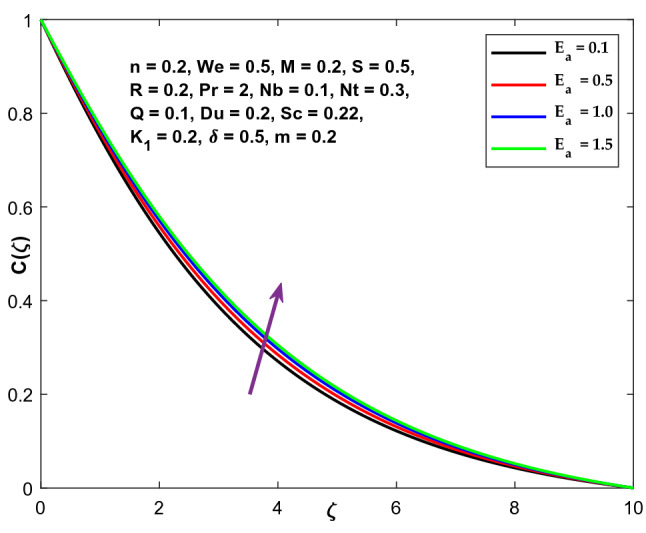
Character of ${E}_{a}$ versus $\mathrm{C}\left(\zeta \right)$.

The effect of activation energy ${E}_{a}$ on volumetric concentration can be examined in Fig. 21. It is noticed that increasing the activation energy ${E}_{a}$ increases the volumetric concentration.

**Figure 22:**
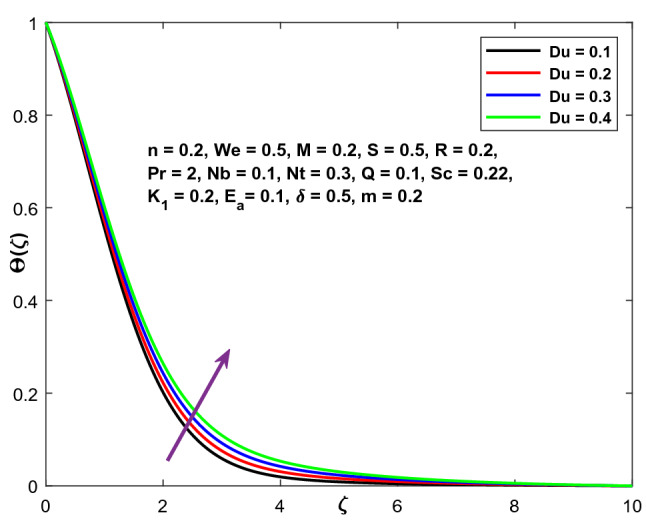
Character of ${E}_{a}$ versus $\mathrm{C}\left(\zeta \right)$.

Figure 22. illustrates Dufour's influence on the temperature field. It has been observed that raising the Du number results in an increase in the temperature field.

**Figure 23:**
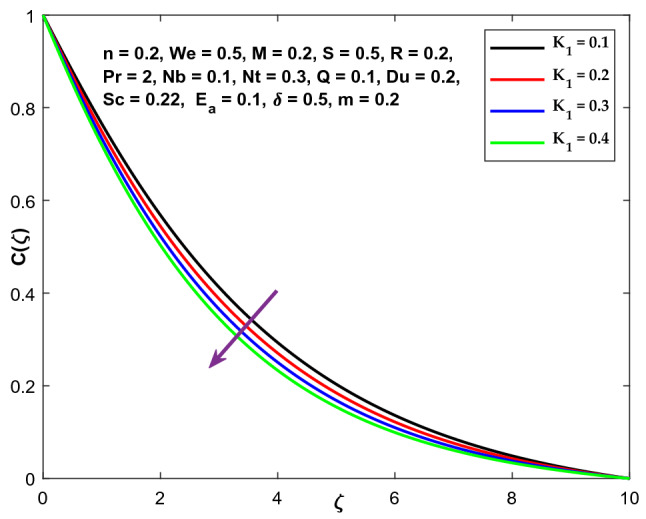
Character of ${K}_{1}$ versus $\mathrm{C}\left(\zeta \right)$.

The fluctuation of a chemical reaction factor on a concentration profile is shown in Fig. 23. It demonstrates that the concentration profile diminishes as the value of ${K}_{1}$ enhances.

## Validation of numerical scheme

The $- \Theta^{\prime } (0)$ comparison values are used to validate the numerical data. Table 1 compares^1–3^. As a result of the excellent agreement between the numerical results, we may be sure of the results' trustworthiness.

**Table 1:** Comparison results of $\Theta^{\prime } (0)$ in the absence of the $n,We,M,R,Nb, Nt,Q, Du$.

*Pr*	Hassanien et al.^40^	Salleh and Nazar^41^	Fadzilah et al.^42^	Present study
0.72	0.46325	0.46317	0.4632	0.46321
1	0.58198	0.58198	0.582	0.58198
3	1.16525	1.16522	1.1652	1.16522
7	–	1.89548	1.8954	1.89548
10	2.30801	2.30821	2.3081	2.30820

The intention of Table 2 is to evaluate the effect of relevant factors on the skin friction coefficient. Notably, the positive value of modified magnetic number M, the power-law index n, and the Weissenberg number diminish the surface drag coefficient.

**Table 2:** Variation of $-{C}_{f}\sqrt{{Re}_{x}}$ when $S = 0.5, \;R = 0.2, \;Pr = 2, \;Nb = 0.1, \; Nt = 0.3, \; Q = 0.1, \; Du = 0.2, \; Sc = 0.22, \; Sr = 0.5, \; K1 = 0.2,\; Ea = 0.1, \; \delta = 0.5, \; m = 0.2.$

n	M	We = 0	We = 0.1	We = 0.3
0	0	1.000008	1.000008	1.000008
0.1	0	0.948688	0.946636	0.942484
0.2	0	0.894429	0.889788	0.880228
0.3	0	0.836661	0.828639	0.811668
0.4	0	0.774597	0.761964	0.733972
	0.5	0.573424	0.571065	0.566277
	1	0.289674	0.288642	0.286570
	1.5	0.023972	0.023555	0.022719

Table 3 demonstrates the effect of different variables on the Nusselt number. The heat transfer rate is lowered when the power-law coefficient n, thermophoresis coefficient Nt, the heat source (Q), and the Weissenberg number (We) values improve. However, the Nusselt number grows as the thermal radiation parameter (R) rises.

**Table 3:** Variation of $N{u}_{x}{/\sqrt{Re}}_{x}$ when $M=0.2, \; S = 0.5, \; R = 0.2, \; Pr = 2, \; Nb = 0.1, \; Du = 0.2, \; Sc = 0.22, \; K1 = 0.2, \; Ea = 0.1, \; \delta = 0.5, \; m = 0.2.$

$Nt$	$n$	$We$	$Nb$	$R$	$Q$	$N{u}_{x}{/\sqrt{Re}}_{x}$
0.1	0.2	0.1	0.1	0.1	0.1	0.726866
0.2						0.662243
0.3						0.604184
0.1	0.3					0.713256
	0.4					0.696965
	0.5					0.676964
	0.2	0.2				0.725883
		0.3				0.724879
		0.4				0.723852
		0.1	0.2			0.695927
			0.3			0.664049
			0.4			0.631435
			0.1	0.2		0.763673
				0.3		0.796755
				0.4		0.826666
				0.1	0.2	0.574271
					0.3	0.375493
					0.4	0.069734

Table 4 shows the influence of various factors on the mass transfer rate or the Sherwood number. It is observed that there is an acclivity in each of the power-law index n, the Weissenberg number. (We), Activation energy ($E_{a}$), the rate of heat transfer is decreased. In contrast, for increasing values of thermal exponent term (m), heat basis constant ($\delta$), chemical reaction constant($K_{1}$), and Schmidt number, an increase in the Sherwood number is seen.

**Table 4:** Variation of $S{h}_{x}{/\sqrt{Re}}_{x}$ when $M =0.2, \; S=0.5, \; R=0.1, \; Pr=2, \; Nb=0.1, \; Nt=0.1, \; Q=0.1, \; Du=0.1, \; n=0.2$.

$Sc$	$n$	$We$	$m$	*δ*	${E}_{a}$	${K}_{1}$	$S{h}_{x}{/\sqrt{Re}}_{x}$
0.22	0.2	0.1	0.1	0.1	0.1	0.1	0.228192
1.25							0.707772
1.6							0.823877
0.22	0.3						0.224501
	0.4						0.220483
	0.5						0.216056
	0.2	0.2					0.227984
		0.3					0.227773
		0.4					0.227559
		0.1	0.2				0.228192
			0.3				0.228260
			0.4				0.228331
			0.1	0.2			0.228086
				0.3			0.228123
				0.4			0.228158
				0.1	0.2		0.227782
					0.3		0.227406
					0.4		0.227062
					0.1	0.2	0.233026
						0.3	0.237775
						0.4	0.242445

## Conclusions

In this scientific study, the numerical simulation on EMHD transmission of non-Newtonian hyperbolic tangent nanoliquid across a stretching sheet surface with Dufour effect, heat generation, and activation energy is studied. Utilizing the MATLAB software bvp4c, the overview of the outcomes is as follows:

- The improvement of Modified Hartmann number (M) on velocity distribution has a concurrent reverse effect on power-law index (n), Weissenberg number (We), and associated EMHD parameter (S).
- Increasing the values of Du parameter (Du), thermophoresis, and Brownian motion parameters results in the rise of temperature distribution.
- The augmentation of the activation energy ${E}_{a}$ increases the volumetric concentration.
- The increasing values of modified magnetic number M, the power-law index (n), and the Weissenberg number slow down the friction coefficient.
- The rate of heat transfer is lowered when the thermophoresis parameter (Nt), the power-law index n, heat source (Q), and the Weissenberg number (We) increases

## Data Availability

The numerical data used to support the findings of this study are included within the article.

## List of symbols

$M$: Modified Hartmann parameter
$$T$$: Fluid temperature
$$We$$: Weissenberg number
$$Nu$$: Nusselt number
$$T_{\omega }^{{}}$$: Surface temperature
$$E_{a}$$: Activation energy
$\delta$: Heat basis constant
$$Du$$: Dufour effect
$n$: Power-law index
$$\,C_{\omega }^{{}}$$: Surface concentration
$K_{1}$: Chemical reaction constant
$\,m$: Thermal exponent term
$Q$: Heat generation parameter
$$Sc$$: Schmidt number
l: Magnets and electrodes width
$C_{p}$: Specific heat capacity
$$Nb$$: Brownian motion
$$k^{*}$$: Mean absorption
$$Nt$$: Thermophoresis parameter
$$T_{\infty }^{{}}$$: Ambient temperature
$$Sh$$: Sherwood number
$$C_{s}$$: Concentration susceptibility
$D_{m}$: Coefficient of mass diffusivity
$m_{0}$: Magnetization of magnets (Tesla)
$$\Pr$$: Prandtl number
$\sigma^{*}$: Stefan Boltzmann constant
$S$: Associated with magnets and electrodes
$$R$$: Radiation parameter
$$\rho$$: Density
$q_{r}$: Radiative heat flux
$C_{\infty }^{{}}$: Ambient fluid concentration
$$k$$: Thermal conductivity
